# Repurposing Olive Oil Mill Wastewater into a Valuable Ingredient for Functional Bread Production

**DOI:** 10.3390/foods14111945

**Published:** 2025-05-29

**Authors:** Ignazio Restivo, Lino Sciurba, Serena Indelicato, Mario Allegra, Claudia Lino, Giuliana Garofalo, David Bongiorno, Salvatore Davino, Giuseppe Avellone, Luca Settanni, Luisa Tesoriere, Raimondo Gaglio

**Affiliations:** 1 Department of Biological, Chemical and Pharmaceutical Science and Technology (STEBICEF), University of Palermo, Via Archirafi, 90123 Palermo, Italy; ignazio.restivo@unipa.it (I.R.); serena.indelicato@unipa.it (S.I.); mario.allegra@unipa.it (M.A.); claudia.lino@unipa.it (C.L.); david.bongiorno@unipa.it (D.B.); luisa.tesoriere@unipa.it (L.T.); 2 Department of Agricultural, Food and Forest Sciences, University of Palermo, Viale delle Scienze, Bldg. 5, 90128 Palermo, Italy; lino.sciurba@unipa.it (L.S.); giuliana.garofalo01@unipa.it (G.G.); salvatore.davino@unipa.it (S.D.); raimondo.gaglio@unipa.it (R.G.)

**Keywords:** functional bread, in vitro digestion, lactic acid bacteria, olive wastes, polyphenols, sourdough

## Abstract

Untreated olive oil mill wastewater (OOMW) from conventionally farmed olives was used in bread production to create a new functional product. Two types of bread were developed with 50% OOMW (EXP-1) and 100% OOMW (EXP-2) replacing water. Two leavening processes were tested: sourdough inoculum (*S*) vs. biga-like inoculum (*B*), with controls (CTR) without OOMW addition. The doughs were monitored throughout the acidification process by measuring pH, total titratable acidity, and the development of key fermentative microorganisms. To assess the hygienic quality during fermentation, plate count techniques were employed. After baking, the breads were evaluated for various quality parameters, including weight loss, specific volume, crumb and crust colors, image analysis, and the presence of spore-forming bacteria. Volatile compounds released from the breads were identified using solid-phase microextraction coupled with gas chromatography–mass spectrometry (SPME-GC/MS). Polyphenolic compounds were analyzed via liquid chromatography–mass spectrometry (LC-MS). To assess the functional properties of the final products, the breads were homogenized with synthetic human saliva and subjected to in vitro digestion. OOMW did not significantly affect the growth of yeasts and lactic acid bacteria (LAB) or the acidification process. However, in terms of the specific volume and alveolation, breads from the *S* process and OOMW had poor quality, while those from the *B* process had better quality. Experimental breads (EXP*_B_*-1 and EXP*_B_*-2) contained higher levels of alcohols (especially ethanol and isobutyl alcohol), carbonyl compounds (like benzaldehyde), esters (such as ethyl caproate and ethyl caprylate), and terpenes. OOMW introduced phenolic compounds like hydroxytyrosol, coumaric acid, caffeic acid, and trans-hydroxycinnamic acid, which were absent in CTR*_B_* breads. Functionalization of EXP*_B_*-1 and EXP*_B_*-2 breads was demonstrated by a 2.4- and 3.9-fold increase in Trolox equivalents, respectively. However, OOMW did not reduce post-prandial hyper-glycemia, as starch digestibility was similar between CTR*_B_* and EXP*_B_* breads. The sensory analysis, which focused solely on the visual, structural, and olfactory characteristics of the breads, excluding taste testing to prevent potential health risks from residual pesticides, showed a high appreciation for EXP*_B_*-1 and EXP*_B_*-2 breads, scoring higher than CTR*_B_* in the overall assessment.

## 1. Introduction

Despite numerous efforts in recent years to manage wastes from agricultural activities and food by-products, these residues continue to pose environmental challenges and incur disposal costs for producers [1,2]. Over the past decade, numerous global institutions have called for more sustainable policy choices to address these issues, promoting the circular economy model as a strategy for achieving greater global sustainability [3]. Ensuring the sustainability of the food production process remains one of the most critical challenges for the future [4].

In this context, the olive oil industry holds substantial economic importance within the European Union (EU), producing over 6–7 million tons of oil annually [5]. Olive cultivation is widespread in the Mediterranean basin, with Italy being one of the top producers [6]. However, the olive sector generates substantial waste. The extraction process results in three by-products: olive paste, the fleshy part of the olive fruit remaining after oil extraction; olive pomace, the primary solid residue left after oil extraction; and olive oil mill wastewater (OOMW), the liquid waste produced during oil production [7]. While olive paste and olive pomace are typically reused as soil fertilizers or combustible raw materials [8], OOMW is more challenging to recycle due to its strong odor, dark brown color, and acidic pH [9]. Nevertheless, OOMW is rich in bioactive compounds like polyphenols [10], which are known for their health benefits [11]. According to a review by Obied et al. [12], it contains over 30 biophenols and related compounds with antioxidant properties and potential cardioprotective and cancer-preventive effects.

Given the growing need for more efficient and sustainable approaches, innovative non-conventional methods are gaining traction. These methods utilize low-cost raw materials to extract antioxidant compounds, which can be applied in food supply chains, as well as in the nutraceutical and cosmetics sectors. Additionally, the rising consumer demand for nutritionally rich and functional foods has caught the attention of food producers. In this context, cereals and cereal-based products are ideal candidates for fortification, as they are a staple in the daily diet and provide a significant amount of macronutrients and micronutrients, including vitamins and minerals. However, it is important to note that thermal treatments, such as baking and mechanical processes like milling, can significantly reduce or even eliminate dietary fiber and bioactive compounds, potentially diminishing the nutritional value of these products. Therefore, OOMW could be a promising by-product to enhance the positive properties of bread. In addition to its richness in bioactive compounds, it boosts dietary antioxidant levels, often diminished during baking, and introduces unique phytochemicals absent in conventional formulations. Its use supports the development of health-promoting foods, aligns with sustainability and circular economy principles by repurposing polluting agro-industrial waste, and reduces disposal costs due to its high organic load and phytotoxicity. Moreover, OOMW contributes to a more complex and appealing aroma profile through its volatile compounds.

Sourdough fermentation is a traditional and widely used technology in Italian bread making [13]. This process primarily relies on the activity of lactic acid bacteria (LAB), particularly, the heterofermentative species [14]. While yeasts also play a crucial role, especially in leavening, the presence of LAB is essential for developing the bread’s characteristic aroma and extending its shelf life [15]. Despite typically resulting in lower loaf volume and reduced crumb porosity compared to baker’s yeast fermentation, sourdough technology offers several advantages, including improved flavor and preservation [16]. In addition to sourdough, many Italian-baked products, such as bread and pizza, are also commonly produced using biga technology [17]. This method involves a pre-fermentation step with a small amount of yeast, which is allowed to ferment slowly at room temperature before being incorporated into the final dough [18].

This investigation aimed to create new functional bakery products by incorporating OOMW. In a previous study by Sciurba et al. [19], the same bulk of OOMW was analyzed for pesticide residues and was found to contain four active compounds: Azoxystrobin (0.2 μg/L), Bromacil (5.29 μg/L), Imidacloprid (0.17 μg/L), and Simazine (0.21 μg/L). Regarding the toxicological implications of the detected pesticide residues in OOMW, Bromacil is mildly irritating to the eyes and skin but does not exhibit neurotoxic, genotoxic, reproductive, developmental, or immunotoxic effects; Simazine has been associated with fetal developmental delays, endocrine disruption, and mammary tumors; Azoxystrobin shows weak genotoxic responses in mammalian cells but is considered unlikely to pose a carcinogenic risk to humans; and Imidacloprid may cause symptoms such as drowsiness, dizziness, vomiting, disorientation, and fever. The effects of this incorporation were assessed by monitoring the acidification process, key rheological parameters, and the polyphenol and volatile organic compound contents of the final products. Fermentation was carried out using two different approaches: a sourdough starter and a biga-like inoculum. Additionally, in vitro digestion tests and sensory evaluations (without taste tests), which are crucial for launching a new product onto the market, were conducted.

## 2. Materials and Methods

### 2.1. Raw Materials and Fermenting Bacteria

A 10 L sample of olive oil mill wastewater (OOMW) was collected in a plastic container for food materials (Ecoplast S.r.l., Gela, Italy) shortly after milling at a facility in Partinico (Palermo province, Italy). Within 6 h, it was delivered to the Food and Agricultural Microbiology lab at the University of Palermo (Italy) using a portable cooler to prevent microbial growth. Upon arrival at the laboratory, the OOMW was transferred into 1 L sterile Durham bottles and frozen at −20 °C. The composition of OOMW consisted of approximately 84% (*v*/*v*) aqueous solution, 12.5% (*v*/*v*) pomace, and 3.5% (*v*/*v*) residual oil.

In this study, *Leuconostoc mesenteroides* RC-UNIPASAAFM01342 (previously strain OMW 23) and *Lactiplantibacillus plantarum* RC-UNIPASAAFM01341 (previously strain OMW 1), isolated from OOMW and intrinsically resistant to olive polyphenols [19], were added to *Fructilactobacillus sanfranciscensis* RC-UNIPASAAFM01100, *Weissella cibaria* RC-UNIPASAAFM01109, and *Leuconostoc citreum* RC-UNIPASAAFM01118, originating from sourdoughs used in Sicilian bakeries and part of the SAAF Department’s culture collection at the University of Palermo, and were employed to start the sourdough fermentation.

### 2.2. Sourdough Propagation

**Figure 1:**
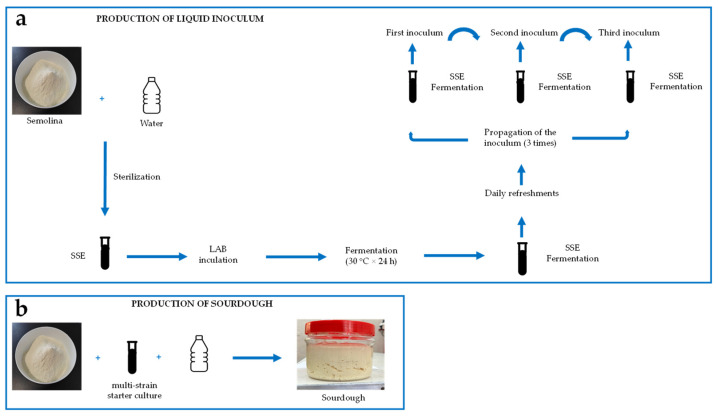
Sourdough production: (**a**) pre-fermentation process to prepare the liquid inoculum; (**b**) development of sourdough with the multi-strain starter culture.

To prepare sourdough inoculums, all strains were defrosted from glycerol stocks stored at −80 °C and cultured twice in a modified de Man-Rogosa-Sharpe (mMRS) medium following the procedure outlined by Corsetti et al. [20]. Subsequently, the bacteria were incubated for about 24 h at 30 °C before being inoculated into a mixture with semolina to develop the sourdough. All LAB strains were inoculated individually and propagated in liquid form using a sterile semolina extract (SSE) as the growth medium, as it can support growth beyond 10^9^ CFU/mL [21]. All LAB strains were sub-cultivated three times and combined to prepare a multi-strain starter culture [22] (Figure 1a).

The cell suspension was diluted with sterile tap water to reach a final volume of 187.5 mL. This mixture was then combined with 312.5 g of semolina (La Molisana S.p.A., Ripalimosani, Italy) to form a 500 g dough. The dough had a dough yield (DY = weight of the dough/weight of semolina × 100) of 160 and a cell density of approximately 10^6^ to 10^7^ CFU/g. The dough underwent fermentation at 28 °C for 16 h and was refreshed daily over a period of seven days, following the method outlined by Corona et al. [23], to produce a mature sourdough suitable for baking (Figure 1b).

### 2.3. Dough Production and Baking

Two different processes were conducted to test the suitability of OOMW in bread production. The first process was carried out using only the sourdough (trials *S*) developed in this study as a leavening agent, while in the second process, commercial baker’s yeast was added to the sourdough to create a biga-like dough (trials *B*). For both processes (*S* and *B*), three 800 g tests with a DY of 175 were performed: CTR, control production; EXP-1, experimental 1 (50% OOMW in substitution of water); and EXP-2, experimental 2 (100% OOMW in substitution of water).

For the *S* process, the control production (CTR*_S_*) was prepared with the following recipe: 457.2 g of semolina, 228.6 mL of sterile tap water, and 114.2 g of the 7-day matured sourdough. The experimental tests were prepared as follows: EXP*_S_*-1 with 457.2 g of semolina, 114.3 mL of sterile tap water, 114.3 mL of OOMW, and 114.2 g of sourdough; EXP*_S_*-2 with 457.2 g of semolina, 228.6 mL of OOMW, and 114.2 g of sourdough. In the *B* process, 8 g of commercial baker’s yeast (Conad, Bologna, Italy) were added to each of the three recipes described above for CTR*_S_*, EXP*_S_*-1, and EXP*_S_*-2 to prepare CTR*_B_*, EXP*_B_*-1, and EXP*_B_*-2 productions.

A planetary mixer model XBM10S (Electrolux Professional, SpA, Pordenone, Italy) was used to blend all ingredients at speed 4 for 15 min. To better assess the impact of OOMW on the stability of LAB inoculums, no salt was included in the formulation. For each trial, 6 doughs were prepared, making a total of 18 doughs for the *S* process and 18 for the *B* process. The doughs (100 g each) were placed in stainless steel trays conforming to the trapezoidal dimensions indicated by the American Association of Cereal Chemists—AACC Method 10-10B [24]. The trays were covered with aluminium foil during fermentation, which was conducted at 28 °C for 8 h for the *S* process and at 28 °C for 2 h for the *B* process. The rest of the doughs (200 g from each batch) were placed into sterile glass jars (Vetreria Borgonovo S.p.A., Borgonovo Val Tidone, Italy) to allow fermentation under controlled sterile conditions. The baking process took place in a semi-industrial Compact Combi oven (Electrolux, Pordenone, Italy) using a two-phase program based on the manufacturer’s guidelines: the doughs were initially subjected to hot air/steam treatment at 200 °C for 5 min, followed by 15 min of convective heating at the same temperature.

### 2.4. Acidification Process

Fermentation of the sourdough and the doughs intended for baking was monitored by measuring the pH, total titratable acidity (TTA), and the evolution of fermenting microorganisms. Both parameters were measured immediately after inoculation and during fermentation. Measurements were conducted at intervals of 0, 2, 4, 6, and 8 h for the S process (with sourdough inoculum) and at 0 and only after the first 2 h for the B process (with biga-like dough inoculum).

To measure pH, the probe of the pH meter (XS Instruments, Carpi, Italy) was directly inserted into the SSE test tubes containing 10 mL of sample or into 10 g of solid samples, such as sourdough, biga-like dough, and doughs prepared for baking, all collected under sterile conditions. Simultaneously, TTA measurements were taken. The same samples used for pH assessment were then placed into stomacher bags, combined with 90 mL of distilled water, and homogenized using a stomacher BagMixer^®^ 400 (Interscience, Saint Nom, France) at its highest speed for 2 min. Following this, titration was performed with a 0.1 N NaOH solution. TTA values were reported as the volume of 0.1 N NaOH required per 10 g of dough.

To determine the viable count of fermenting groups, all samples were homogenized as previously described, but sterile Ringer’s solution (Sigma-Aldrich, Milan, Italy) was used in place of distilled water. The resulting homogenates were then subjected to serial decimal dilutions and inoculated on various media: plate count agar (PCA) for total mesophilic microorganisms (TMM), incubated aerobically at 30 °C for 72 h; mMRS agar for mesophilic rod-shaped lactic acid bacteria (LAB), incubated anaerobically at 30 °C for 48 h; on sourdough bacteria (SDB) agar for sourdough LAB [25], incubated aerobically at 30 °C for 48 h; and yeast peptone dextrose (YPD) agar with chloramphenicol (0.1 g/L) for yeasts, incubated at 28 °C for 48 h. Additionally, Enterobacteriaceae were enumerated on violet red bile glucose agar (VRBGA), incubated at 37 °C for 24 h, and total coliforms on violet red bile agar (VRBA), also incubated at 37 °C for 24 h. All culture media were purchased from Oxoid. Microbiological analyses were performed in duplicate, and results were expressed as log CFU/g.

### 2.5. Bread Quality Parameter Determination

After baking, all bread samples were allowed to cool at room temperature for 30 min prior to quality assessment. Weight loss (WL) due to water evaporation during baking was calculated using the formula:

$$WL=\left(\frac{wd-wb}{wd}\right)\times 100$$

where “wd” represents the weight (g) of the dough and “wb” represents the weight (g) of the baked bread [26].

Bread volume was measured using a 2 L volumeter designed for bakery products, with flax seeds placed in the appropriate container (ErreCi srl, Merate, Italy).

The color of the bread crust and crumb was assessed using a Chroma Meter CR-400 (Minolta, Osaka, Japan) based on the Hunter’s scale parameters (L*, a* and b*) [27]. Specifically, three points on the crumb from the central slice and four points on the upper surface of each bread were analyzed.

Bread hardness was evaluated by measuring compression resistance (N/mm^2^) using an Instron-5564 (Instron Corp., Canton, MA, USA), following the method described by Corsetti et al. [28].

The central slice of each bread was analyzed using image processing techniques. Each slice was examined for void fraction (percentage of the total area occupied by alveoli), cell density (number of alveoli/cm^2^), and the average area of the alveoli (in mm^2^). The analysis was conducted after scanning the bread slices at a resolution of 350 dpi using an Epson Perfection 4180 Photo scanner (Seiko Epson Corp., Suwa, Japan). The scanned images, saved in TIFF format, were processed with ImageJ software (National Institutes of Health, Bethesda, MD, USA). Each image was cropped into squares of 207 × 207 pixels (representing an area of the bread slice of 15 × 15 mm), converted to 8-bit grayscale, and binary images were generated using Otsu’s thresholding method.

Additionally, the bread samples were tested for the presence of aerobic spore-forming bacteria. Each bread sample (25 g) was serially diluted in Ringer’s solution, following the same procedure used for the dough samples. The cell suspensions were then heated to 85 °C for 15 min, and 0.1 mL aliquots were spread on Nutrient Agar (NA) plates (Oxoid, Basingstoke, UK), which were incubated at 32 °C for 48 h [29].

### 2.6. Volatile Organic Compound Analysis by SPME-GC/MS

Volatile compounds were analyzed using solid-phase microextraction (SPME) followed by GC/MS. Six grams of sample were equilibrated at 60 °C for 30 min in a sealed 40 mL vial. A DVB/CAR/PDMS fiber (50/30 µm, Supelco, Bellefonte, PA, USA) was exposed to the headspace for 30 min and then, thermally desorbed at 230 °C for 5 min in the GC injector. Fiber pre-conditioning was performed at 270 °C for 1 h prior to analysis. Analyses were conducted using a Trace 1310 GC coupled to a TSQ 800 triple quadrupole MS (Thermo Fisher Scientific, Milan, Italy). Volatiles were separated on a TG-XLBMS column (20 m × 0.18 mm i.d., 0.18 μm film thickness). Helium was used as carrier gas at 1.0 mL/min. The oven temperature was maintained at 35 °C for 5 min, then ramped up to 100 °C at a rate of 5 °C/min, held at this temperature for 2 min, and then increased to 180 °C at a rate of 6 °C/min and maintained for 2 min. The temperature was then further increased to 230 °C at the rate of 8 °C/min and held for an additional 2 min. (details in Table S1). Electron ionization (EI) was performed at 70 eV with full scan acquisition (30–350 *m*/*z*). Compound identification was based on NIST library matching and/or authentic standards. Analyses were performed in triplicate.

### 2.7. Analysis of Polyphenolic Compounds by LC-MS/MS

Polyphenols were extracted from 2 g of sample using 5 mL methanol/water (80:20 *v*/*v*), followed by vortexing, sonication, and centrifugation. The supernatant was filtered (0.45 μm PTFE) and diluted 1:100 with methanol. A 5 µL aliquot was injected into an Ultimate 3000 LC system coupled to a TSQ Quantiva triple quadrupole MS (Thermo Fisher Scientific). Chromatographic separation was performed on a Hypersil GOLD C18 column (2.1 × 50 mm, 1.9 µm) at 30 °C, using a gradient elution with water (0.1% formic acid) and methanol. MS detection was carried out in negative ion mode with an HESI source. Selected Reaction Monitoring (SRM) transitions for 19 target polyphenols are reported in Table S2. Quantification was based on external calibration curves (from 1 ppm to 5 ppb) with R^2^ > 0.99. LODs, and LOQs were determined from the blank signal and the regression curve.

### 2.8. Determination of Functional Properties of Breads

Two grams of bread samples were homogenized using a Waring blender (Waring, New Hartford, CT, USA) equipped with a semi-micro stainless-steel jar in 10 mL of a pH 6.8 buffered solution designed to replicate the chemical composition of human saliva according to European Pharmacopoeia. The aqueous suspension was submitted to in vitro digestion following the standardized INFOGEST 2.0 method [22]. To separate the bioaccessible fraction from the undigested material, the post-intestinal mixture was subjected to ultracentrifugation at a force of 167,000× *g* for 35 min at 4 °C, using a Beckman Optima TLX ultracentrifuge (Beckman Instruments, Inc., Palo Alto, CA, USA). This process yielded a supernatant consisting of the bioaccessible fraction isolated from the residual nonabsorbable particulate material.

Bread samples were subjected to in vitro digestion following a previously established protocol. During the intestinal phase, 1 mL aliquots of the digesta were collected at intervals of 0, 20, 40, 60, 80, and 120 min after the addition of pancreatin. These aliquots were immediately transferred to tubes placed in boiling water for 4 min to stop enzymatic activity. Following ultracentrifugation, the glucose content in the supernatant was measured spectrophotometrically at 510 nm using a D-glucose assay kit (GOPOD Format, Megazyme International, Ltd., Bray, Ireland). The starch digestibility was expressed as mg of glucose per gram of initial starch.

One milliliter of the aqueous suspension of bread, both before digestion and from the bio-accessible fractions obtained post in vitro digestion, was centrifuged at 1000× *g* for 15 min at 4 °C. This process effectively separated the soluble components from any particulate matter. The resulting soluble fractions were then processed to determine total phenolic content and assess radical scavenging activity.

For total phenol content determination, aliquots of 100 µL from each sample, prepared at three different dilutions to ensure accuracy across a range of concentrations, were mixed with 3 mL of 2% sodium carbonate (Na_2_CO_3_) solution [30]. Then, 100 µL of the Folin–Ciocalteu reagent, pre-diluted in a 1:1 ratio with distilled water, was added to initiate the reaction. This mixture was left to incubate in the dark at room temperature for 60 min to allow color development. The absorbance of the resulting solutions was measured at 765 nm using a Beckman DU 640 spectrophotometer (Beckman Instruments, Milan, Italy). A blank solution was used as a reference to zero the instrument. The phenolic content in each sample was determined by measuring absorbance and comparing it to calibration curves created using gallic acid standards, with concentrations ranging from 5 to 100 µg/mL. The results were expressed as milligrams of gallic acid equivalents per gram of bread.

The radical-scavenging capacity of the bread was assessed through the decolorization assays of the 2,2′-azino-bis (3-ethylbenzothiazoline-6-sulfonic acid) (ABTS) radical cation and of the 2,2-diphenyl-1-picrylhydrazyl (DPPH) radical. The ABTS•+ radical was generated by oxidizing ABTS with potassium persulfate and diluted with 5 mM phosphate saline buffer to obtain an absorbance of 0.700 ± 0.020 units at 734 nm [31]. Aliquots of properly diluted bread solution were added, and the absorbance registered again after 15 min.

The 2,2-diphenyl-1-picrylhydrazyl (DPPH) free radical scavenging activity was determined following the method described by Attanzio et al. [32]. For the assay, 10 μL of bread solution was mixed with 1 mL of DPPH ethanol solution at a concentration of 1 × 10^−4^ mol/L. The mixture was incubated at room temperature in the dark for 30 min, after which the absorbance was measured at 515 nm.

To ensure precision, each sample was analyzed in duplicate and at three different dilutions within the assay’s linear range. Calibration was performed using Trolox, a water-soluble analog of vitamin E, and the antioxidant activity was expressed as Trolox equivalents (TE), measured in μmol of Trolox equivalents per gram of bread.

### 2.9. Sensory Analysis

Only the final breads from the B process were evaluated through a descriptive sensory analysis by a panel of 13 trained judges, comprising 8 women and 5 men, aged between 23 and 55 years. The panelists were instructed to assess various attributes of the bread, including appearance, structure, and aroma, following the methodologies outlined by Comendador et al. [33], Martins et al. [34], and Rodrigues et al. [35]. Each attribute was rated on a 9-point scale, where 1 represented extremely poor and 9 represented extremely good. Additionally, the judges provided an overall evaluation based on the combined scores of all attributes. The sensory analysis was conducted in individual booths in accordance with ISO 13299 [36] standards. Taste parameters were excluded from the evaluation due to the presence of pesticide residues in the OOMW [19], which precluded tasting the breads.

### 2.10. Statistical Analysis

A one-way analysis of variance (ANOVA) was conducted to detect differences in the microbiological and physicochemical data. Tukey’s test was subsequently employed for multiple mean comparisons, with statistical significance established at *p* < 0.05. All statistical analyses were performed using XLStat version 7.5.2 for Excel (Addinsoft, New York, NY, USA).

## 3. Results and Discussion

### 3.1. Fermentation Process

The fermentation process initiated by the selected LAB strains, used as starters, was monitored from the preparation of the liquid inoculum in SSE. The growth of these strains in the substrate resulted in a reduction in the initial pH from 5.7 to an average of 3.63 ± 0.1. The highest pH value (3.72 ± 0.1) was observed for the *Ln. mesenteroides* RC-UNIPASAAFM01342 strain, while the lowest pH (3.55 ± 0.2) was recorded for *Lp. plantarum* RC-UNIPASAAFM01341. These pH changes are consistent with those typically seen in LAB (mainly lactobacilli, leuconostoc, and weissella) grown in flour and semolina extracts [15]. The five fermented SSE samples, at the third propagation phase, were combined to form the liquid inoculum used to produce the sourdough for bread leavening [15]. The sourdough developed with these five LAB strains had an initial pH of 3.7 ± 0.01 and a TTA value of 16.8 ± 0.02 mL NaOH 0.1 N/10 g. These acidification parameters (pH and TTA) were similar to those reported by Alfonzo et al. [21] and Gaglio et al. [22]. After seven days of daily refreshments, the sourdough was deemed mature and ready for use.

#### 3.1.1. Evolution of Fermentation Parameters for *S* Process

Table 1 presents the pH and TTA measurements for the experimental doughs from the *S* process. At the initial time point (T0), the control dough (CTR*_S_*) exhibited a slightly higher pH compared to the doughs with added OOMW. This lower pH in the experimental doughs can be attributed to the OOMW itself, which had a pH close to 5.0 due to the presence of organic acids [37]. This parameter decreased across all samples, with the CTR sample exhibiting the lowest pH of 3.84 by the end of fermentation. The TTA value evolves inversely to the pH; this acidification parameter increases linearly as the pH decreases and is due to the production of organic acids during fermentation, mainly lactic acid. By the end of fermentation, the TTA values for the EXP*_S_* doughs (10.4 mL NaOH 0.1 N/10 g for both EXP*_S_*-1 and EXP*_S_*-2) were higher (<0.0001) than those of the CTR*_S_* doughs, (9.5 mL NaOH 0.1 N/10 g), indicating a greater buffering effect of the OOMW pH.

The microorganisms of greatest interest during the fermentation of the doughs were enumerated and reported in Table 2. At T0, LAB levels (~7 log CFU/g) were consistent across samples and similar to TMM (7.30–7.45 log CFU/g), indicating LAB dominance [38]. By T8, LAB increased by ~2 logs only in CTR*_S_*, while remaining stable in EXP*_S_*-1 and EXP*_S_*-2. LAB counts were slightly higher than TMM at both time points, likely due to limited nutrients in PCA. Another important microbial group in sourdough fermentation is yeasts [39]. Their levels rose from 4.08–4.42 log CFU/g to just below 6 log CFU/g, consistent with spontaneous growth in sourdough [40]. Members of the Enterobacteriaceae family and total coliforms are used as hygiene indicators [41]. Enterobacteriaceae, which are part of the microbiome of durum wheat semolina [42], present at T0 in CTR and dropped to near zero by T8, likely due to low pH [43].

#### 3.1.2. Evolution of Fermentation Parameters for *B* Process

The pH and TTA measurements of the experimental doughs from the *B* process are presented in Table 3. After fermentation, the pH dropped in all samples, with CTR*_B_* being the highest (5.09). The TTA increased, especially in EXP*_B_*-2 (11 mL NaOH 0.1 N/10 g).

Table 4 presents the main populations influencing the fermentation of the *B* process. TMM was ~7 log CFU/g at T0, rising in all samples by 2 h, and peaking in CTR*_B_* (8.36 log CFU/g). LAB levels at T0 ranged from 7.15 to 7.78 log CFU/g; after 2 h, they rose by ~1 log cycle in CTR*_B_*, with a smaller increase in EXP*_B_*-1 and EXP*_B_*-2 doughs. Yeast counts were similar across groups at T0 (7.34–7.52 log CFU/g) and increased by ~1 log by T2 (8.22–8.42 log CFU/g), with no significant differences (*p* > 0.05). Enterobacteriaceae in CTR*_B_* dropped from 1.60 log CFU/g at T0 to near 0, likely due to low pH [43].

### 3.2. Quality Characteristics of Final Breads

Table 5 summarizes bread quality from the *S* and *B* processes. In the *S* process, baking led to weight losses of 13.27–14.09%, with specific volume decreasing as OOMW increased (2.67 cm^3^/g in CTR*_S_* to 2.16 cm^3^/g in EXP*_S_*-2). OOMW addition altered crust and crumb color and the lightness increased, while redness and yellowness decreased; the a* (red) parameter increased significantly (*p* < 0.05) and was found among the three treatments, showing a constant increase.

**Figure 2:**
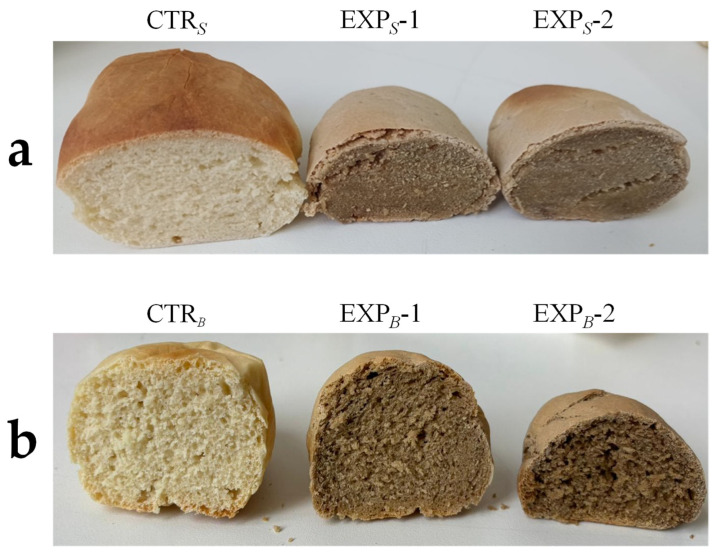
Breads processed with OOMW. (**a**) *S* process breads, produced using exclusively sourdough as fermentation agent. Abbreviations: CTR*_S_*, control production; EXP*_S_*-1, experimental 1 production (50% OOMW in substitution of water); EXP*_S_*-2, experimental 2 production (100% OOMW in substitution of water). (**b**) *B* process breads, produced using a biga-like fermentation agent. Abbreviations: CTR*_B_*, control production; EXP*_B_*-1, experimental 1 production (50% OOMW in substitution of water); EXP*_B_*-2, experimental 2 production (100% OOMW in substitution of water).

Image analysis revealed that increasing the percentage of OOMW in the bread led to a higher void fraction and cell density in the crumb, while the alveolation decreased. These data highlighted the production of OOMW breads with unacceptable quality characteristics, particularly in relation to their compactness and very limited alveolation (Figure 2a).

In the *B* process, OOMW reduced the peak height and specific volume (3.50 to 2.50 cm^3^/g) of the ciabatta bread, while increasing baking weight loss. However, commercial yeast improved leavening. Therefore, the biga-like developed with the help of *Saccharomyces cerevisiae,* allowing for better leavening compared to that obtained with only sourdough. Color parameters were significantly affected, with lower lightness in CTR*_B_* breads and notable a* parameter differences in the crumb (*p* < 0.05). Image analysis of the central slices of the breads revealed differences between the CTR*_B_* breads and those containing OOMW across all three alveolation-related parameters. This analysis showed higher void fraction and alveolar area in *B* process breads due to yeast (Figure 2b). Given the better quality in the *B* process breads, further analyses focused on CTR*_B_*, EXP*_B_*-1, and EXP*_B_*-2 breads.

Finally, the investigation for aerobic spore-forming bacteria yielded no positive findings, indicating that this group was absent in all the bread samples analyzed.

### 3.3. Volatile Organic Compounds

More than 60 total volatile compounds were recognized from *B* process breads, which constitute the aromatic component of all samples analyzed. For each sample, they were grouped by chemical class: acids, alcohols, carbonylic compounds, esters, furan compounds, terpenes, hydrocarbons, and ethers (Table 6). For each compound, the relative abundance was evaluated, a quantitative approach that has limitations, as the areas of each peak do not reflect the real quantities of the different compounds, but, these percentages are very useful as a comparison tool and provide useful indications to evaluate the contribution of OOMW in this case for bread.

The volatile aromatic components of the breads were analyzed using HS-SPME-GC/MS. In the CTR*_B_* bread, 44 compounds belonging to different classes were identified, including 1 acid, 6 alcohols, 15 carboxylic compounds, 7 esters, 4 terpenes, 8 hydrocarbons, and 1 ether. Yeasts, which are crucial in bread production, facilitate the synthesis of various volatiles, such as alcohols, aldehydes, acids, esters, and ketones, through fermentation [44]. The most relevant alcohols identified were ethanol, 3-methyl-butanol, and phenylethyl alcohol generated during fermentation or from the Maillard reaction [22]. Interestingly, 3-methyl-butanol imparts balsamic, floral, and malty notes to bread and correlates with the flavor of wheat crumb and that phenylethyl alcohol is known to have fruity, honey, lilac, and rose odors [44]. Among carbonyl compounds, hexanal, benzaldehyde, and nonal contribute to the aromatic component of the control bread and may derive from lipid oxidation. The compound 2-pentyl furan is produced through thermal reactions, such as the Maillard reaction and caramelization, which contribute to the formation of the crust and its characteristic aroma [45,46]. Limonene is also present in the control bread sample, which is generally associated with naturally leavened bread [23].

Additionally, it is noteworthy that these samples contain several compounds absent in the control bread, suggesting they are specifically introduced by adding OOMW. Key compounds include 2-octen-1-ol, 1-octanol, 2-octenal, copaene, and farnesene. Moreover, the relative area percentages of certain compounds increased from the EXP*_B_*-1 sample to the EXP*_B_*-2 sample, indicating that the addition of OOMW had a significant impact on the volatile aroma profile of the breads. Specifically, from the EXP*_B_*-1 sample to the EXP*_B_*-2 sample, 3-methyl-butanol increased from 10.76% to 18.47%, 1-hexanol from 2.46% to 3.89%, phenylethyl alcohol from 3.60% to 4.49%, benzaldehyde from 2.74% to 3.66%, and both ethyl caproate and hexanal showed slight increases from 2.66% to 2.99% and from 0.97% to 1.08%, respectively.

**Table 6:** Volatile aromatic compounds expressed as a percentage of three replicates expressed as (peak area of each compound/total area of significant peaks) × 100, and odor descriptors identified in *B* process breads, produced using a biga-like fermentation agent.

n°	CAS	Chemical Compounds	Samples			Aroma	Odor Threshold μg/kg in Water	Reference
			CTR*_B_*	EXP*_B_*-1	EXP*_B_*-2			
		Acids						
1	64-19-7	Acetic acid	1.65 ± 0.76	n.d.	n.d.	pungent, sour	22,000–320,000	[47]
		Alcohols						
2	64-17-5	Ethanol *	20.34 ± 0.00	42.62 ± 0.17	42.55 ± 0.66	sweet	100,000	[48]
3	18,409-17-1	2-Octen-1-ol (E) *	n.d.	0.11 ± 0.01	0.13 ± 0.01	green	1	[48]
4	111-87-5	1-Octanol *	n.d.	0.19 ± 0.00	0.14 ± 0.01	bitter almond, burnt matches, fat, floral	110–130	[48]
5	123-51-3	3-methyl-butanol	19.81 ± 0.04	9.36 ± 2.4	18.81 ± 0.95	balsamic, floral, malt	250–300	[48]
6	111-27-3	1-Hexanol	2.55 ± 0.32	2.80 ± 0.86	3.96 ± 0.16	banana, flower, grass, herb	2500	[48]
7	78-27-3	Cyclohexanol, 1-ethynyl-	0.41 ± 0.30	n.d.	n.d.	odorless		[48]
8	104-76-7	1-Hexanol, 2-ethyl	0.69 ± 0.22	0.31 ± 0.02	0.29 ± 0.03	rose, green	-	[48]
9	60-12-8	Phenylethyl Alcohol *	8.55 ± 0.00	4.09 ± 0.93	4.58 ± 0.46	fruit, honey, lilac, rose, wine	750–1100	[48]
10	40,642-37-3	Z-4-dodecenol	0.41 ± 0.11	0.41 ± 0.11	0.27 ± 0.05	waxy		[48]
11	513-85-9	2,3-Butanediol	n.d.	1.49 ± 2.02	n.d.	fruit, onion	-	[48]
12	78-83-1	Isobutyl alcohol	n.d.	n.d.	1.95 ± 0.75	apple, bitter, cocoa, wine	7000	[49]
13	4798-44-1	3-Hexen-1-ol	n.d.	n.d.	0.58 ± 0.04	green	70	[48]
14	123-92-2	Isoamyl acetate	n.d.	n.d.	0.22 ± 0.03	apple, banana, pear (fruity)	2	[49]
		Carbonylic Compounds						
15	66-25-1	Hexanal	2.39 ± 1.09	1.10 ± 0.68	1.10 ± 0.03	apple, fat, fresh, green, oil	4.5–5	[48]
16	111-71-7	Heptanal *	2.20 ± 0.23	0.52 ± 0.18	0.28 ± 0.01	citrus, fat, green, nut	3	[48]
17	100-52-7	Benzaldehyde	2.58 ± 0.23	3.12 ± 0.29	3.73 ± 0.26	bitter almond, burnt sugar, cherry, malt, roasted pepper	350–3500	[48]
18	124-13-0	Octanal	0.44 ± 0.07	0.20 ± 0.01	0.28 ± 0.02	fat, soap, lemon, green	0.7	[48]
19	36,431-60-4	5-Ethylcyclopent-1-enecarboxaldehyde	0.44 ± 0.01	0.48 ± 0.10	0.26 ± 0.01	corn flavor		[48]
20	122-78-1	Benzeneacetaldehyde	0.48 ± 0.10	0.43 ± 0.02	0.69 ± 0.11	berry, geranium, honey, nut, pungent	4	[48]
21	124-19-6	Nonanal *	2.59 ± 0.16	1.72 ± 0.03	1.40 ± 0.25	fat, floral, green, lemon	1	[48]
22	60,784-31-8	2-Nonenal (E)	0.58 ± 0.10	0.79 ± 0.00	0.48 ± 0.05	orris, fat, cucumber	0.08–0.1	[48]
23	2548-87-0	2-Octenal (E) *	n.d.	0.86 ± 0.07	0.36 ± 0.01	dandelion, fat, fruit, grass, green, spice	3	[48]
24	112-31-2	Decanal	0.50 ± 0.14	0.24 ± 0.08	0.16 ± 0.02	floral, fried, orange peel, penetrating, tallow	0.1–2	[48]
25	110-43-0	2-Heptanone *	0.43 ± 0.03	0.09 ± 0.02	0.08 ± 0.00	penetrating fruity odor	140–3000	[48]
26	110-93-0	5-Hepten-2-one, 6 methyl	0.51 ± 0.24	0.39 ± 0.00	0.37 ± 0.06	powerful, fatty, green, citrus	50	[49]
27	1669-44-9	3-Octen-2-one	0.25 ± 0.05	0.18 ± 0.05	0.12 ± 0.02	dull, green, nut, rose	-	[44]
28	3796-70-1	Geranylacetone	0.13 ± 0.03	0.10 ± 0.02	n.d.	floral and fruit	60	[49]
29	484-31-1	Apiol	0.02 ± 0.00	0.06 ± 0.01	0.01 ± 0.00	wood, spice		
		Esters						
30	142-92-7	Hexyl Acetate *	n.d.	0.27 ± 0.00	0.16 ± 0.04	sweet-fruity, pearl-like odor	2	[48]
31	123-66-0	Ethyl caproate	1.51 ± 0.33	3.02 ± 0.56	3.04 ± 0.58	fruity	1	[48]
32	106-30-9	Ethyl heptanoate	0.60 ± 0.15	0.90 ± 0.00	0.60 ± 0.06	brandy, fruit, wine	2.2	[49]
33	106-32-1	Ethyl caprylate	4.78 ± 0.26	7.59 ± 0.52	4.49 ± 0.04	fruit, fat	-	[48]
34	103-45-7	Phenethyl acetate	0.19 ± 0.05	0.32 ± 0.07	0.21 ± 0.01	flower, honey, rose		
35	123-29-5	Ethyl Nonanoate *	0.12 ± 0.00	n.d.	0.09 ± 0.00	floral	-	[48]
36	110-38-3	Ethyl caprate	0.71 ± 0.03	0.57 ± 0.11	0.29 ± 0.00	brandy, grape, pear	-	[48]
37	106-33-2	Ethyl laurate	0.01 ± 0.00	0.01 ± 0.00	n.d.	floral, fruit, leaf	400	[50]
38	539-82-2	Ethyl valerate	n.d.	n.d.	0.07 ± 0.03	apple, dry fish, herb, nut, yeast	1.5–5	[49]
39	106-70-7	Methyl caproate	n.d.	n.d.	0.07 ± 0.03	ester, fresh, fruit, pineapple	70–84	[48]
		Furan Compounds						
40	3777-69-3	Furan,2-pentyl-	6.50 ± 1.12	4.46 ± 0.05	0.79 ± 0.13	butter, floral, fruit, green bean	6	[48]
		Terpenes						
41	99-87-6	o-Cymene	0.35 ± 0.11	0.17 ± 0.03	0.09 ± 0.04	solvent, gasoline, citrus	-	[48]
42	5989-27-5	D-Limonene	4.05 ± 1.60	2.47 ± 0.28	1.39 ± 0.51	citrus, mint	10	[48]
43	502-99-8	α-cis-Ocimene	1.63 ± 0.81	0.88 ± 0.20	0.59 ± 0.20	fruit, wet cloth		
44	87-44-5	Caryophyllene	0.12 ± 0.09	0.11 ± 0.03	0.06 ± 0.01	fried, spice, wood	64	[49]
45	3856-25-5	Copaene	n.d.	0.40 ± 0.10	0.47 ± 0.04	wood, spice		
46	502-61-4	α-Farnesene *	n.d.	0.22 ± 0.01	0.17 ± 0.00	boiled vegetable, floral, wood		
		Hydrocarbons						
47	111-84-2	Nonane *	2.29 ± 0.41	0.77 ± 0.00	0.79 ± 0.25	gasoline-like odor		
48	13,475-82-6	Isododecane	0.61 ± 0.24	0.36 ± 0.00	0.38 ± 0.14	odorless		
49	41,898-89-9	2,3-Heptadien-5-yne 2,4-dimethyl	1.33 ± 0.53	0.66 ± 0.18	0.49 ± 0.11	odorless		
50	112-40-3	Dodecane	1.65 ± 1.19	1.44 ± 0.87	0.92 ± 0.38	gasoline-like to odorless		
51	629-50-5	Tridecane	0.61 ± 0.16	0.77 ± 0.28	0.28 ± 0.02	gasoline-like to odorless		
52	629-59-4	Tetradecane	1.11 ± 0.48	0.64 ± 0.25	0.32 ± 0.00	gasoline-like to odorless		
53	1632-70-8	Undecane, 5-methyl	n.d.	0.07 ± 0.03	0.06 ± 0.00	odorless		
54	61,141-72-8	Dodecane, 4,6-dimethyl-	n.d.	0.12 ± 0.11	n.d.	odorless		
55	51,655-65-3	2-butyl-1-decene	n.d.	0.09 ± 0.04	0.07 ± 0.00	odorless		
56	6117-97-1	Dodecane, 4-methyl	n.d.	0.05 ± 0.04	n.d.	odorless		
57	54,833-48-6	Heptadecane, 2,6,10,15-tetramethyl	0.41 ± 0.18	0.06 ± 0.01	n.d.	odorless		
58		3-Ethyl-1,5-octadiene	n.d.	n.d.	0.13 ± 0.00	odorless		
59	629-62-9	Pentadecane	0.11 ± 0.02	n.d.	n.d.	oil of d. guineense fruit		
60	128-37-0	Butylated Hydroxytoluene	3.22 ± 0.89	1.85 ± 0.32	1.20 ± 0.02	very faint, musty, occasional cresylic-type odor		
		Ether						
61	629-82-3	Octyl ether	0.17 ± 0.07	0.06 ± 0.01	0.02 ± 0.00	odorless		

Abbreviations: CTR*_B_*, control production; EXP*_B_*-1, experimental 1 production (50% OOMW in substitution of water); EXP*_B_*-2, experimental 2 production (100% OOMW in substitution of water); n.d., not detected. All the compounds marked with * of EXP1*_B_*-1 and EXP*_B_*-2 showed values significantly different (at *p* 0.05%) from trial CTR*_B_.*

### 3.4. Polyphenolic Profiles

Several targeted phenolic compounds were detected and quantified from the *B* process breads (Table 7). As an example, SRM chromatograms of EXP*_B_*-2 breads are shown in Figure S1. Table 7 clearly shows that, when the CTR*_B_* bread is compared to the EXP*_B_*-1 and EXP*_B_*-2 breads, only 5 out of the 19 investigated polyphenols (Table S2) were detected, with varying concentrations. However, it is evident that the major contribution comes from hydroxytyrosol, which is the most abundant polyphenol among the four and is clearly derived from OOMW. Following in decreasing order are trans-hydroxycinnamic acid, caffeic acid, and coumaric acid. Notably, these latter compounds are found exclusively in the enriched bread samples, confirming that the flour used for baking does not contribute to their presence. Thus, it is clear that the addition of vegetation waters plays a crucial role in increasing polyphenol content, making the final product potentially a functional food. Furthermore, the increase in OOMW percentage correlates with a progressive rise in total polyphenol content, reaching 7.11 mg/100 g in EXP*_B_*-1 bread and 10.27 mg/100 g in EXP*_B_*-2 bread. Finally, it is worth noting that oleacin and ferulic acid, which were present in OOMW at the lowest concentrations [19], are no longer detectable in the final product.

### 3.5. Functional Aspects

It is well established that only 2% of the total phenolic content of milled olive fruit transfers to the oil phase, approximately 53% goes to the OOMW, and the remaining amount to the pomace [51]. Then, OOMW represents a valuable reservoir of organic compounds that have high radical scavenger activity and health-promoting properties. The OOMW used in this study contained 18.56 ± 1.35 mg GAE/mL of total phenols, as determined by the Folin–Ciocalteu assay. The antioxidant potential of OOMW was higher in the ABTS test (30.68 ± 2.56 μmol TE/mL) than DPPH test (9.64 ± 0.83 μmol TE/mL).

Oxidative stress concurs in the development and progression of various human diseases, such as diabetes, atherosclerosis, and even cancer. Correct nutrition is one of the most important prevention approaches because diet antioxidants, including phenolic compounds, can contribute to human health by scavenging free radicals [52]. To assess the functional properties of the fortified bakery product, we evaluated the radical-scavenging ability of breads containing OOMW produced using the *B* process (EXP*_B_*-1 and EXP*_B_*-2), in comparison to the CTR*_B_* bread. Given that olive oil phenols can be unstable under digestive conditions [53], we measured their quantity and anti-radical activity in bread samples both before and after simulated gastrointestinal digestion.

**Figure 3:**
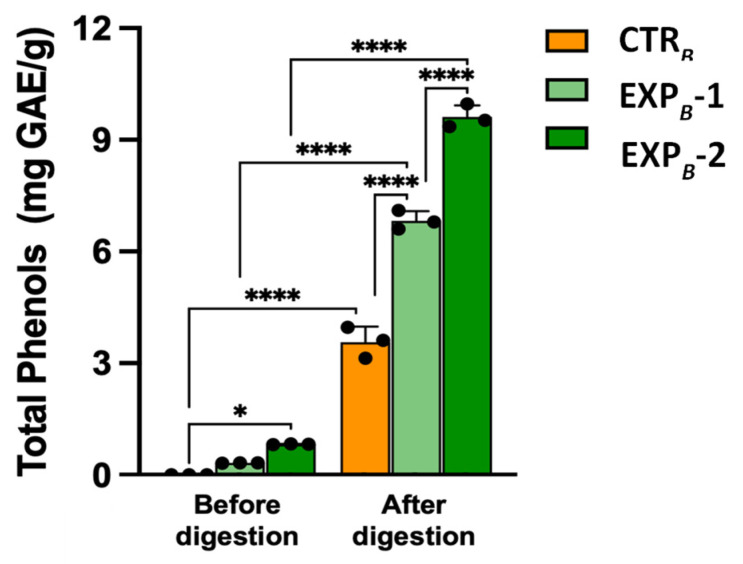
Total phenol released from *B* process breads before and after in vitro digestion. Values are the mean ± SD *n* = 3 experiments. * *p* < 0.05; **** *p* < 0.0001 (ANOVA associated with Tukey’s test). Abbreviations: CTR*_B_*, control production; EXP*_B_*-1, experimental 1 production (50% OOMW in substitution of water); EXP*_B_*-2, experimental 2 production (100% OOMW in substitution of water).

Phenols released from EXP*_B_*-1 and EXP*_B_*-2 breads before digestion were measured at 0.32 ± 0.04 mg GAE/g and 0.83 ± 0.05 mg GAE/g (*n* = 4), respectively (Figure 3). Considering the addition of 0.2 mL/g and 0.4 mL/g of OOMW to EXP*_B_*-1 and EXP*_B_*-2, respectively, and that semolina has a content of phenolic compounds of 0.06 mg GAE/g (CTR*_B_* bread), it turns out that about 10% of the phenols can be solubilized. Indeed, as reported by Dahdah et al. [54], interactions between the polyphenols of the olive oil and the starch-gluten matrix of the semolina greatly limit their release. Instead, post-intestinal bioaccessible fractions obtained after in vitro simulated digestion of the OOMW-enriched breads showed a tenfold increase in phenol content compared to the relevant samples before digestion (Figure 3), with an almost 100% release of the phytochemicals.

**Figure 4:**
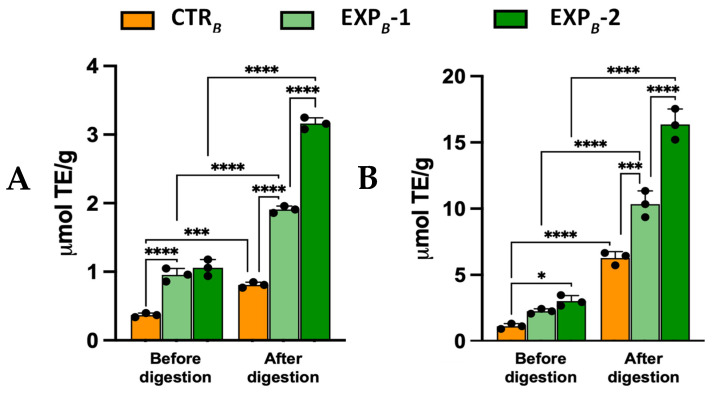
Radical reducing activity of *B* process breads towards DPPH (**A**) and ABTS+• (**B**). Values are the mean ± SD *n* = 3 experiments. * *p* < 0.05; *** *p* < 0.001; **** *p* < 0.0001 (ANOVA associated with Tukey’s test). Abbreviations: CTR*_B_*, control production; EXP*_B_*-1, experimental 1 production (50% OOMW in substitution of water); EXP*_B_*-2, experimental 2 production (100% OOMW in substitution of water).

The DPPH decoloration assay revealed 2.5-fold and 1.8-fold higher activities in the EXP*_B_*-1 and EXP*_B_*-2 breads vs. the CTR*_B_* bread, increasing their radical scavenging capacity by, respectively, before digestion (Figure 4A). Post-digestion values reached 1.91 ± 0.11 and 3.16 ± 0.28 μmol TE/g in EXP*_B_*-1 and EXP*_B_*-2 breads, respectively, which is 2.4-fold and 3.9-fold higher than CTR*_B_* bread (Figure 4A). Similar results were obtained using the ABTS•+ decolorization assay (Figure 4B). These findings demonstrate that the digestion process of bread fortified with OOMW facilitates the release of active compounds in the digested fraction, thereby enhancing the antioxidant potential of the product and improving its functional health properties.

**Figure 5:**
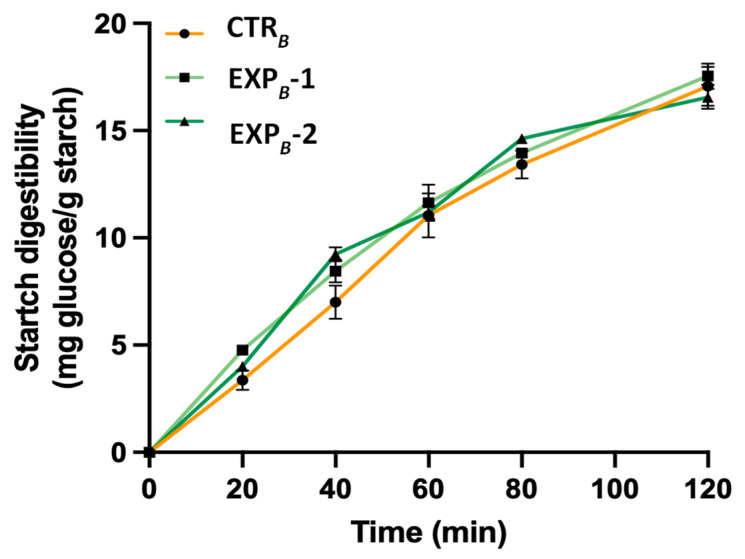
Starch digestibility during in vitro intestinal digestion of *B* process breads. Data are the mean ± SD of *n* = 3 experiments. (ANOVA associated with Tukey’s test). Abbreviations: CTR*_B_*, control production; EXP*_B_*-1, experimental 1 production (50% OOMW in substitution of water); EXP*_B_*-2, experimental 2 production (100% OOMW in substitution of water).

The starch digestibility assessment during in vitro digestion of carbohydrate-rich products is considered a valid method to predict the potential in vivo glycemic response of the product [55]. We measured the glucose release from the breads during in vitro pancreatic digestion to study how the incorporation of phenolic compounds affects starch digestibility. The findings revealed that enriching bread with polyphenols derived from OOMW did not significantly affect starch digestibility compared to the control bread, suggesting that the enriched baked product is not able to reduce post-prandial hyperglycemia (Figure 5). This result should not be surprising. In fact, although it has been reported that breads baked with olive oil can attenuate the glycaemic response in humans, the formation of amylose-fatty acids complexes and not the presence of secondary metabolites appears responsible for the observed effect [56].

### 3.6. Sensory Attributes

**Figure 6:**
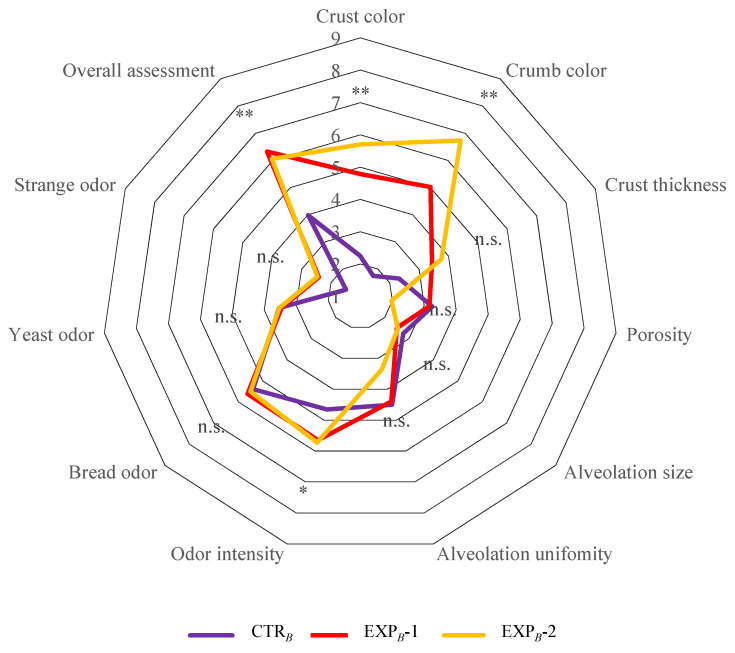
Spider diagrams of descriptive sensory analysis of *B* process breads. Abbreviations: CTR*_B_*, control production; EXP*_B_*-1, experimental 1 production (50% OOMW in substitution of water); EXP*_B_*-2, experimental 2 production (100% OOMW in substitution of water). *, *p* < 0.01; **, *p* < 0.001; n.s., not significant.

The sensory analysis of the breads was limited to the evaluation of appearance, texture, and smell, as the chemical data of OOMW did not allow for taste evaluation [13]. In general, the color of the crumb and crust, the thickness of the crust, and the intensity of the smell increased with the percentage of OOMW, while the porosity and alveolation decreased. It is important to note that, in the overall evaluation, the bread from the EXP*_B_*-1 and EXP*_B_*-2 trials was particularly appreciated. This preference is undoubtedly attributable to the pleasant hint of olive oil and, more generally, it resembles olive bread, justifying the higher preference compared to the control bread (CTR*_B_*) (Figure 6). This result is particularly relevant, as it confirms through a sensory evaluation panel, although limited by the absence of taste examination, the results shown by Perito et al. [57] on the positive perception of consumers towards the use of by-products from the olive oil industry to develop new food preparation technologies and, consequently, new food products.

## 4. Conclusions

This study demonstrated the potential of untreated OOMW as a functional ingredient in bread making. Breads made with OOMW contained polyphenolic compounds absent in control breads or present at higher concentrations. These experimental breads also exhibited stronger antioxidant activity compared to control breads. Sensory evaluation indicated that these breads were well accepted by panelists. However, sourdough alone was insufficient for producing acceptable breads, and the addition of baker’s yeast was necessary to achieve satisfactory leavening and structure and for future industrial-scale production. Due to concerns over pesticide residues, sensory testing was limited, highlighting the importance of sourcing OOMW from organic farming. While conventionally derived OOMW may pose safety concerns, organic OOMW could offer a viable and sustainable alternative. Once we demonstrate the food suitability of organic OOMW, this approach could create functional breads while addressing environmental concerns related to food production. Reusing OOMW in bread and baked goods supports the circular economy model by repurposing significant agro-industrial waste from the olive processing industry. Hence, future work will focus on characterizing organic OOMW and validating its safety and functionality in food applications.

## Figures and Tables

**Table 1:** Acidification parameters of *S* process doughs, produced using exclusively sourdough as fermentation agent.

Time	Parameter	Samples			*p* Value
		CTR*_S_*	EXP1*_S_*-1	EXP*_S_*-2	
0 h	pH	5.24 ± 0.01 ^a^	5.14 ± 0.01 ^b^	5.14 ± 0.01 ^b^	<0.0001
	TTA	5.2 ± 0.04 ^b^	6.6 ± 0.03 ^a^	6.6 ± 0.03 ^a^	<0.0001
2 h	pH	4.99 ± 0.01 ^b^	5.03 ± 0.02 ^b^	5.08 ± 0.02 ^a^	0.002
	TTA	5.5 ± 0.02 ^c^	7.3 ± 0.01 ^b^	9.8 0.02 ^a^	<0.0001
4 h	pH	4.29 ± 0.01 ^c^	4.65 ± 0.01 ^b^	4.77 ± 0.03 ^a^	<0.0001
	TTA	8 ± 0.02 ^c^	9.1 ± 0.03 ^b^	10.7 ± 0.02 ^a^	<0.0001
6 h	pH	4.07 ± 0.01 ^c^	4.21 ± 0.01 ^b^	4.27 ± 0.01 ^a^	<0.0001
	TTA	9 ± 0.01 ^c^	9.8 ± 0.02 ^b^	10.7 ± 0.02 ^a^	<0.0001
8 h	pH	3.84 ± 0.01 ^b^	4.27 ± 0.02 ^a^	4.29 ± 0.01 ^a^	<0.0001
	TTA	9.5 ± 0.03 ^b^	10.4 ± 0.01 ^a^	10.4 ± 0.1 ^a^	<0.0001

The results indicate the mean values ± S.D. (standard deviation) of the counts performed in duplicate. Values within the same row marked with different letters are significantly different according to Tukey’s test. Abbreviations: CTR*_S_*, control production; EXP*_S_*-1, experimental 1 production (50% OOMW in substitution of water); EXPS-2, experimental 2 production (100% OOMW in substitution of water).

**Table 2:** Microbial loads of *S* process doughs, produced using exclusively sourdough as fermentation agent.

Media	Time	Samples			*p* Value
		CTR_S_	EXP1_S_-1	EXP_S_-2	
PCA	0 h	7.30 ± 0.07 ^a^	7.45 ± 0.21 ^a^	7.34 ± 0.06 ^a^	0.412
	8 h	8.69 ± 0.06 ^a^	7.91 ± 0.08 ^b^	7.89 ± 0.09 ^b^	0.0002
mMRS	0 h	8.29 ± 0.02 ^b^	7.34 ± 0.06 ^c^	7.23 ± 0.08 ^c^	<0.0001
	8 h	9.11 ± 0.04 ^a^	7.88 ± 0.01 ^b^	7.83 ± 0.08 ^b^	<0.0001
SDB	0 h	7.73 ± 0.45 ^a^	7.28 ± 0.15 ^a^	7.27 ± 0.15 ^a^	0.161
	8 h	9.30 ± 0.29 ^a^	7.88 ± 0.05 ^a^	7.75 ± 0.78 ^a^	0.313
YPD	0 h	4.42 ± 0.17 ^a^	4.27 ± 0.38 ^a^	4.08 ± 0.12 ^a^	0.319
	8 h	5.15 ± 0.21 ^a^	5.65 ± 0.06 ^a^	5.84 ± 0.33 ^a^	0.367
VRBA	0 h	1.81 ± 0.23 ^a^	1.43 ± 0.36 ^ab^	<1 ^b^	<0.0001
	8 h	<1	<1	<1	n.d.
VRBGA	0 h	<1	<1	<1	n.d.
	8 h	<1	<1	<1	n.d.

The results indicate the mean values ± S.D. (standard deviation) of the counts performed in duplicate, expressed as log CFU/g. Data within the same row followed by different letters are significantly different according to Tukey’s test. Abbreviations: CTR*_S_*, control production; EXP*_S_*-1, experimental 1 production (50% OOMW in substitution of water); EXP*_S_*-2, experimental 2 production (100% OOMW in substitution of water); n.d., not detected; PCA, Plate Count Agar; mMRS, Modified de Man, Rogosa and Sharpe Agar; SDB, Sabouraud Dextrose Broth; YPD, Yeast Peptone Dextrose Agar; VRBA, Violet Red Bile Agar; VRBGA, Violet Red Bile Glucose Agar.

**Table 3:** Acidification parameters of *B* process doughs, produced using a biga-like fermentation agent.

Time	Parameter	Samples			*p* Value
		CTR*_B_*	EXP1*_B_*-1	EXP*_B_*-2	
0 h	pH	5.23 ± 0.02 ^a^	5.13 ± 0.02 ^a^	5.13 ± 0.03 ^a^	0.173
	TTA	5.7 ± 0.01 ^c^	9 ± 0.03 ^b^	10.4 ± 0.02 ^a^	<0.0001
2 h	pH	5.09 ± 0.01 ^a^	5.02 ± 0.01 ^b^	5.07 ± 0.01 ^a^	<0.0001
	TTA	6.3 ± 0.03 ^c^	10.1 ± 0.01 ^b^	11.00 ± 0.01 ^a^	<0.0001

The results indicate the mean values ± S.D. (standard deviation) of the counts performed in duplicate. Data within the same row followed by different letters are significantly different according to Tukey’s test. Abbreviations: CTR*_B_*, control production; EXP*_B_*-1, experimental 1 production (50% OOMW in substitution of water); EXP*_B_*-2, experimental 2 production (100% OOMW in substitution of water).

**Table 4:** Microbial loads of *B* process doughs, produced using a biga-like fermentation agent.

Media	Time	Samples			*p* Value
		CTR*_B_*	EXP1*_B_*-1	EXP*_B_*-2	
PCA	0 h	7.11 ± 0.10 ^a^	7.17 ± 0.05 ^a^	7.28 ± 0.24 ^a^	0.437
	2 h	8.36 ± 0.26 ^a^	7.64 ± 0.09 ^a^	7.63 ± 0.46 ^a^	0.044
mMRS	0 h	7.78 ± 0.06 ^a^	7.19 ± 0.21 ^b^	7.27 ± 0.12 ^b^	0.005
	2 h	8.33 ± 0.22 ^a^	7.63 ± 0.12 ^b^	7.38 ± 0.15 ^b^	0.001
SDB	0 h	7.25 ± 0.03 ^a^	7.15 ± 0.14 ^a^	7.32 ± 0.12 ^a^	0.232
	2 h	8.10 ± 0.13 ^a^	7.43 ± 0.21 ^c^	7.44 ± 0.09 ^b^	<0.0001
YPD	0 h	7.52 ± 0.10 ^a^	7.34 ± 0.04 ^a^	7.34 ± 0.45 ^a^	0.655
	2 h	8.42 ± 0.01 ^a^	8.22 ± 0.01 ^a^	8.32 ± 0.44 ^a^	0.649
VRBA	0 h	1.60 ± 0.1 ^a^	1.35 ± 0.08 ^ab^	1.45 ± 0.06 ^b^	0.026
	2 h	<1	<1	<1	n.d.
VRBGA	0 h	<1	<1	<1	n.d.
	2 h	<1	<1	<1	n.d.

The results indicate the mean values ± S.D. (standard deviation) of the counts performed in duplicate, expressed as log CFU/g. Data within the same row followed by different letters are significantly different according to Tukey’s test. Abbreviations: CTR*_B_*, control production; EXP*_B_*-1, experimental 1 production (50% OOMW in substitution of water); EXP*_B_*-2, experimental 2 production (100% OOMW in substitution of water); n.d., not detected; PCA, Plate Count Agar; mMRS, Modified de Man, Rogosa and Sharpe Agar; SDB, Sabouraud Dextrose Broth; YPD, Yeast Peptone Dextrose Agar; VRBA, Violet Red Bile Agar; VRBGA, Violet Red Bile Glucose Agar.

**Table 5:** Quality of breads.

*S* Process Breads				
Attributes	Samples			*p* Value
	CTR*_S_*	EXP1*_S_*-1	EXP*_S_*-2	
Weight loss (%)	14.09 ± 0.75 ^a^	13.67 ± 1.11 ^a^	13.27 ± 2.15 ^a^	0.797
Specific volume (cm^3^/g bread)	2.67 ± 0.81 ^a^	2.35 ± 0.1 ^a^	2.16 ± 0.25 ^a^	0.484
Hardness (N/mm^2^)	0.050 ± 0.005 ^a^	0.094 ± 0.037 ^a^	0.103 ± 0.009 ^a^	0.055
Crust				
Lightness (L*)	70.29 ± 5.77 ^a^	71.31 ± 1.53 ^a^	74.29 ± 8.79 ^a^	0.721
Redness (a*)	16.11 ± 1.92 ^b^	7.68 ± 3.58 ^b^	3.69 ± 0.71 ^a^	0.002
Yellowness (b*)	32.16 ± 2.88 ^a^	28.89 ± 1.32 ^a^	26.42 ± 4.09 ^a^	0.139
Crumb				
Lightness (L*)	71.31 ± 1.54 ^a^	73.57 ± 3.17 ^a^	74.84 ± 2.83 ^a^	0.315
Redness (a*)	−1.83 ± 0.19 ^c^	1.30 ± 0.31 ^b^	2.77 ± 0.35 ^a^	<0.0001
Yellowness (b*)	20.81 ± 0.93 ^a^	20.28 ± 0.96 ^a^	19.06 ± 1.21 ^a^	0.189
Void fraction (%)	32.55 ± 6.40 ^b^	42.44 ± 2.92 ^ab^	44.65 ± 4.66 ^a^	0.044
Cell density (n/cm^2^)	121.8 ± 63.41 ^a^	198.87 ± 38.47 ^a^	240.33 ± 35.86 ^a^	0.057
Mean cell area (mm^2^)	0.30 ± 0.14 ^a^	0.21 ± 0.069 ^a^	0.16 ± 0.037 ^a^	0.250
***B* Process Breads**				
	**Samples**			
	**CTR*_B_***	**EXP1*_B_*-1**	**EXP*_B_*-2**	
Weight loss (%)	14.27 ± 0.49 ^a^	13.56 ± 1.30 ^a^	12.87 ± 1.17 ^a^	0.331
Specific volume (cm^3^/g bread)	3.50 ± 1.5 ^a^	2.93 ± 0.06 ^a^	2.50 ± 0.20 ^a^	0.111
Hardness (N/mm^2^)	0.048 ± 0.005 ^a^	0.051 ± 0.004 ^a^	0.053 ± 0.01 ^a^	0.717
Crust				
Lightness (L*)	69.56 ± 7.78 ^a^	70.59 ± 5.35 ^a^	72.41 ± 3.30 ^a^	0.834
Redness (a*)	6.61 ± 2. 79 ^a^	5.11 ± 1.32 ^a^	3.57 ± 0.66 ^a^	0.205
Yellowness (b*)	30.26 ± 7.7 ^a^	25. 71 ± 3.38 ^a^	23.57 ± 2.18 ^a^	0.324
Crumb				
Lightness (L*)	71.61 ± 2.12 ^a^	72.41 ± 3.30 ^a^	73.32 ± 9.15 ^a^	0.936
Redness (a*)	−2.05 ± 0.10 ^b^	3.98 ± 0.15 ^a^	4.71 ± 1.26 ^a^	<0.0001
Yellowness (b*)	23.01 ± 0.56 ^a^	21.92 ± 0.22 ^a^	20.70 ± 4.92 ^a^	0.636
Void fraction (%)	40.69 ± 2.89 ^a^	41.33 ± 4.140 ^a^	46. 45 ± 1.68 ^a^	0.110
Cell density (n/cm^2^)	126.53 ± 86.66 ^a^	138.33 ± 24.53 ^a^	146.07 ± 35.75 ^a^	0.913
Mean cell area (mm^2^)	0.42 ± 0.25 ^a^	0.30 ± 0.90 ^a^	0.27 ± 0.061 ^a^	0.938

The results indicate the mean values ± S.D. (standard deviation) of the counts performed in duplicate. Data within the same row followed by different letters are significantly different according to Tukey’s test. Abbreviations: *S* process breads, produced using exclusively sourdough as fermentation agent; CTR*_S_*, control production; EXP*_S_*-1, experimental 1 production (50% OOMW in substitution of water); EXP*_S_*-2, experimental 2 production (100% OOMW in substitution of water); *B* process breads, produced using a biga-like fermentation agent; CTR*_B_*, control production; EXP*_B_*-1, experimental 1 production (50% OOMW in substitution of water); EXP*_B_*-2, experimental 2 production (100% OOMW in substitution of water).

**Table 7:** UHPLC-MS parameters for phenolic compounds identified in *B* process breads, produced using a biga-like fermentation agent.

Coumpound	mg/100 g		
	**CTR*_B_***	**EXP*_B_*-1**	**EXP*_B_*-2**
Hydroxythyrosol	n.d.	3.39	4.97
Coumaric acid	n.d.	0.74	0.97
Luteolin	n.d.	NF	0.57
Caffeic Acid	n.d.	1.35	1.69
Trans Hydroxy Cynnamic	n.d.	1.62	2.07
	n.d.		
∑ Polyphenols	n.d.	7.10	10.27

Abbreviations: CTR*_B_*, control production; EXP*_B_*-1, experimental 1 production (50% OOMW in substitution of water); EXP*_B_*-2, experimental 2 production (100% OOMW in substitution of water); n.d., not detected. All the values of EXP*_B_*-1 and EXP*_B_*-2 are significantly different (at *p* 0.05%) from trial CTR*_B_*.

## Data Availability

The original contributions presented in the study are included in the article/Supplementary Materials, further inquiries can be directed to the corresponding author.

## Supplementary Materials

The following supporting information can be downloaded at: https://www.mdpi.com/article/10.3390/foods14111945/s1, Figure S1: Selected Reaction Monitoring chromatograms of EXP*_B_*-2 breads, produced using a biga-like fermentation agent and 100% OOMW in substitution of water; Table S1: Conditions employed for the SPME-GC/MS analysis; Table S2: LC-MS/MS parameters for phenolic compounds investigated in bread samples.
