# Supplementary material for: Repurposing Olive Oil Mill Wastewater into a Valuable Ingredient for Functional Bread Production
Source: Foods. 2025 May 29;14(11):1945. doi: 10.3390/foods14111945 (PMC12154094; doi:10.3390/foods14111945)
Supplement: Supplementary file 1 [file foods-14-01945-s001.zip › foods-3650422-supplementary.pdf]

foods

MDPI

Supplementary Materials

Repurposing olive oil mill wastewater into a valuable ingredient for functional bread production

Ignazio Restivo [1], Lino Sciurba [2], Serena Indelicato [1], Mario Allegra [1], Claudia Lino [1], Giuliana Garofalo [2], David Bongiorno [1], Salvatore Davino [2], Giuseppe Avellone [1,*], Luca Settanni [2,*], Luisa Tesoriere [1], Raimondo Gaglio [2]

[1] Department of Biological, Chemical and Pharmaceutical Science and Technology (STEBICEF), University of Palermo, Via Archirafi, 90123, Palermo, Italy
[2] Department of Agricultural, Food and Forest Sciences, University of Palermo, Viale delle Scienze, Bldg. 5, 90128 Palermo, Italy
Correspondence: : beppe.avellone@unipa.it (G. Avellone); luca.settanni@unipa.it (L. Settanni)

Table S1. Conditions employed for the SPME-GC/MS analysis.

**Sample/Matrix**	CTR, control production; EXP-1, experimental 1 (50 % OOMW in substitution of water); EXP-2, experimental 2 (100 % OOMW in substitution of water)
SPME fiber	50/30 µm DVB/CAR/PDMS
Sample equilibration	30 min into a thermostatic water bath at 60 °C
Extraction	30 min into a thermostatic water bath at 60 °C
Column	TG XLBMS column, L=20 m × I.D.=0.18 mm × df= 0.18 µm (Thermo Scientific GC Column)
Injection T	230 °C
Detector	Triple quadruple
Scan range	Full scan, 30-350 dalton
Carrier gas	He 99.9999%, 1.2 mL/min

Table S2. LC-MS/MS parameters for phenolic compounds investigated in bread samples.

	**Precursor Ion (m/z) [M-H]$^-$**	**Product Ion (m/z)**	**Collision Energy (V)**	**RF Lens (V)**
Hydroxytyrosol	153	95	21	97
	153	123	14	97
Cumaric Acid	163	93	31	91
	163	119	13	91
Ferulic Acid	193	134	15	99

	193	178	13	99
Oleacein	319	165	6	122
	319	195	6	122
Oleocanthal	303	165	9	92
	303	285	6	92
Luteolin	285	133	35	187
	285	175	26	187
Apigenin	269	117	35	178
	269	151	25	178
Gallic Acid	169	79	24	101
Mandelic Acid	151	77	18	65
	151	107	10	65
Gentisic Acid	153	108	22	90
	153	109	14	90
Catechin	289	203	20	147
	289	245	15	147
Caffeic Acid	179	107	25	101
	179	135	16	103
Syringic Acid	197	153	12	100
	197	182	14	100
Epicatechin	289	203	20	147
	289	245	15	147
Trans-OH-Cynnamic	163	93	31	90
	163	119	14	90
Rutin	609	271	60	299

	609	300	38	299
Apigenin-7Glu	433	269	20	123
	433	271	20	123
Quercetin	301	151	18	166
	301	179	21	166
Kaempferol	285	202	20	195
	285	239	29	195

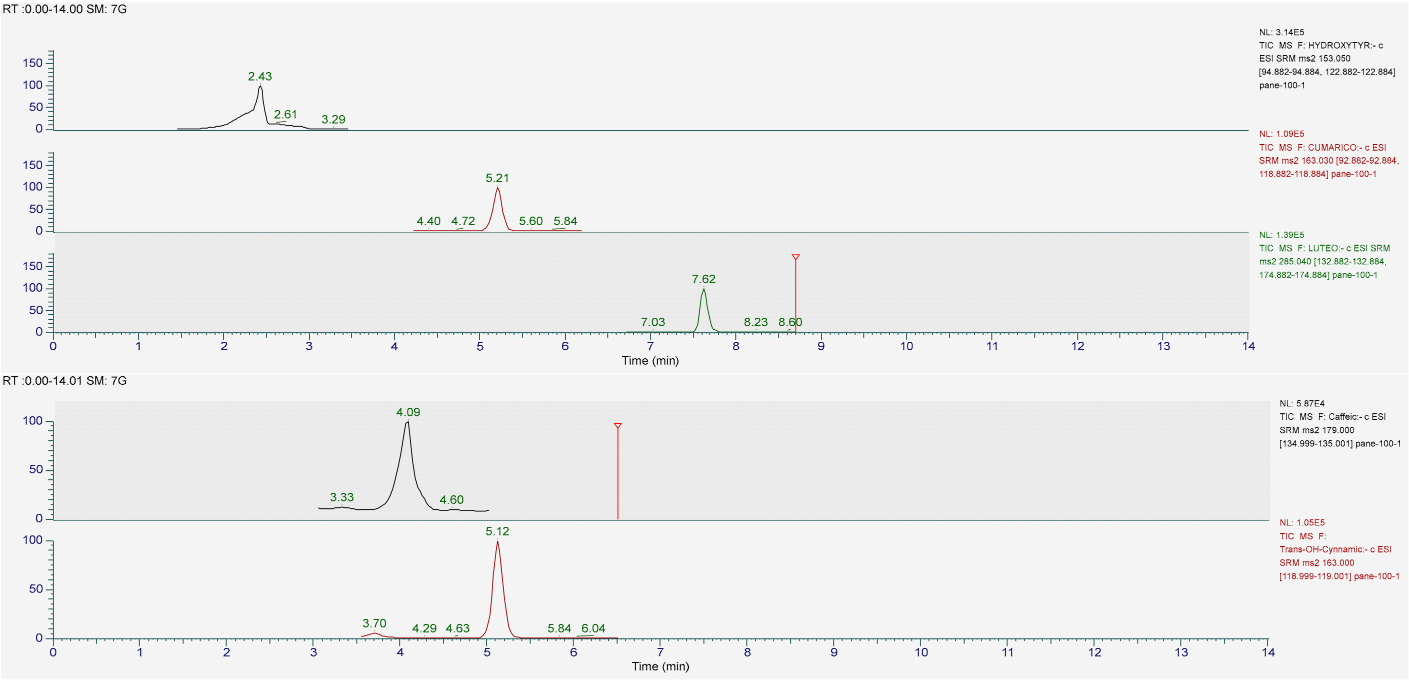

Figure S1. Selected Reaction Monitoring chromatograms of EXP*B*-2 breads, produced using a biga-like fermentation agent and 100% OOMW in substitution of water.
